# Bacteria and Their Products

**Published:** 1891-12

**Authors:** 


*Bacteria and their Products.* By GERMAN SIMS WOODHEAD, M.D. Pp. xiii.—459. London: WALTER SCOTT. 1891.

Bacteriology has so rapidly advanced to a separate science that the time will soon come when medical students will have to recognise micro-organisms and their pure cultivations, just as now they do urine crystals and carcinoma alveoli; and men will not be asked any longer to discuss the various theories of the cause of tetanus, but they will be expected to be familiar with the drum-headed bacillus, and discourse on its nature and habits. And this is the knowledge which in the present day every medical man should have; and we strongly recommend him to study Dr. Woodhead's book, which forms one of the volumes of the "Contemporary Science" series.

It is not so large a work as Flügge's on micro-organisms, but it is very complete, and just published, a point of no small importance in such a rapidly-growing science as bacteriology.

That it is written by Dr. Woodhead is sufficient guarantee of its scientific accuracy; but it is so written that those interested in the subject may read on and on, and feel that from every page they are learning lessons which they can turn to useful account. To medical officers of health the subjects treated of are of the utmost practical importance. The physician, of course, will find very much of interest to him; but the surgeon also, if he turn to such subjects as actinomycosis, tetanus, hydrophobia and others, will not peruse the work in vain.

The book—clearly written and not too condensed—is full of valuable information: one does not read page after page, and then feel inclined to ask oneself how much has been really learned from all this, because here we have no mere summary of conflicting theories, but a collection of worked-out facts. The work is not written wholly for the medical profession. There is a long chapter on fermentation; and although the terms used are not strictly technical medical ones, we think that a great part would not be very intelligible to those unacquainted with medical science. However, an effort is made, as far as possible, to instruct the general reader.

First, we are thoroughly instructed as to the forms and habits of bacilli in general. We are shown how the science has grown, and how, after many futile attempts, pure cultivations were obtained. Then the micro-organisms peculiar to each disease are dealt with, and, in connection with them, all the interesting points relating to their bacteriological origin discussed. At the end of the book is a table, showing how to distinguish various bacilli, which reminds us very much of the analytical tables which taught us in our practical chemistry class how to recognise various chemical substances.

It is impossible, in a short review like this, to go into detail on many points, but we may perhaps refer to the discovery of flagella in an increasing number of bacilli,—the cholera, pneumonia bacillus, and others. Dallinger was the first to discover this thread-like propeller, as we may call it, at the end of a bacillus; and as other bacilli had the same kind of motion, it seemed probable they had the flagella also, and more recently this has been found to be so.

Tuberculosis is a subject of general interest, and is very well dealt with in this book, not only from a pathological, but also from a prophylactic, aspect. Here is a marked example of the practical value of bacteriology. It is very useful to learn that steam and boiling water are some of the most useful agents in rendering inert the tubercle bacillus, because it is surely a simple enough thing to boil clothes and handkerchiefs infected with the sputum of phthisical patients, and to sterilise blankets and other articles by steam. If we cannot cure by tuberculin, the discovery of the bacillus has at least taught us the vast importance of regarding the sputum as infective in nature, to be received in special vessels, and never allowed to dry on clothes or about rooms. In the Grand Duchy of Baden the diminution in the death-rate from phthisis due to strict prophylactic measures, between 1882 and 1887, was from 3.08 per 1000 to 2.8.

But in connection with pathological questions there is also much of great value. The important question of the relation of bacillary invasion to the condition of the tissues is considered, and the explanation is given why caseous material, which seldom contains bacilli, infects with tubercle; and it is surely a very interesting fact to learn that tubercle bacilli are killed by a few days' exposure to sunlight.

We cannot, perhaps, end this review better than by saying that we think this work will become one of the most popular text-books of our pathological series.

